# Artificial hybridization techniques in small millets—A review

**DOI:** 10.3389/fpls.2023.1112117

**Published:** 2023-05-09

**Authors:** T. E. Nagaraja, Nandini C., Sujata Bhat, Gazala Parveen S.

**Affiliations:** ^1^Project Coordinating Unit on Small Millets, Indian Council of Agricultural Research – All India Coordinated Research Project (ICAR-AICRP) on Small Millets, University of Agricultural Sciences, Gandhi Krishi Vignana Kendra (GKVK), Bengaluru, India; ^2^Zonal Agricultural and Horticultural Research Station (ZAHRS), Babbur Farm, Hiriyur, Keladi Shivappa Nayaka University of Agricultural and Horticultural Sciences, Shivamogga, India

**Keywords:** emasculation, hybridization, partial sterile lines, pollination, recombination, small millets

## Abstract

Small millets are nutri-rich, climate-resilient food and fodder crops. They include finger millet, proso millet, foxtail millet, little millet, kodo millet, browntop millet, and barnyard millet. They are self-pollinated crops and belong to the family Poaceae. Hence, to widen the genetic base, the creation of variation through artificial hybridization is a prerequisite. Floral morphology, size, and anthesis behavior cause major hindrances in recombination breeding through hybridization. Manual emasculation of florets is practically very difficult; therefore, the contact method of hybridization is widely followed. However, the success rate of obtaining true F_1_s is 2% to 3%. In finger millet, hot water treatment (52°C) for 3 to 5 min causes temporal male sterility. Chemicals such as maleic hydrazide, gibberellic acid, and ethrel at different concentrations aid in inducing male sterility in finger millet. Partial-sterile (PS) lines developed at the Project Coordinating Unit, Small Millets, Bengaluru are also in use. The percent seed set in crosses derived from PS lines ranged from 27.4 to 49.4, with an average of 40.10%. In proso millet, little millet, and browntop millet, apart from contact method, hot water treatment, hand emasculation, and the USSR method of hybridization are also followed. A newly developed modified crossing method known as the Small Millets University of Agricultural Sciences Bengaluru (SMUASB) method in proso and little millets has a success rate of 56% to 60% in obtaining true hybrids. Hand emasculation and pollination under the greenhouse and growth chamber in foxtail millet with a success rate of 75% seed set is suggested. In barnyard millet, hot water treatment (48°C to 52°C) for 5 min followed by the contact method is often practiced. Kodo millet being cleistogamous, mutation breeding is widely followed to create variation. Most commonly, hot water treatment is followed in finger millet and barnyard millet, SMUASB in proso, and little millet. Although no specific method is suitable for all small millets, it is essential to identify a trouble-free technique that produces maximum crossed seeds in all the small millets.

## Introduction

Small millets belong to the group of staple food grains and fodder crops in semi-arid regions and are popularly known as nutri-cereals. Ecologically, millets are important food crops owing to their short growing season and malleability to adapt to a wide range of temperatures, moisture regimes, and low-input conditions (Tonapi et al., 2022). The commonly cultivated species of small millets include finger millet (*Eleusine coracana* (L.) Gaertn.), kodo millet (*Paspalum scrobiculatum* L.), proso millet (*Panicum miliaceum* L.), foxtail millet (*Setaria italica*), little millet (*Panicum sumatrance*), barnyard millet (*Echinocloa frumentacea*), and browntop millet (*Brachiaria ramose* L.). In India, an area of 4.44 lakh hectares is under the cultivation of small millets, with a total production of 3.46 lakh tonnes and a productivity of 7.81 quintals/hectare (Anonymous, 2021). In recent years, small millets have gained immense significance across the world for their potential to combat hunger and malnutrition and ensure food and nutritional security for the masses. The United Nations General Assembly recently adopted a resolution proposed by India and backed by more than 70 other countries designating the year 2023 as the International Year of Millets.

A total of 248 varieties of small millets are released for commercial cultivation across India, of which 121 are in finger millet, 32 in foxtail millet, 24 in proso millet, 33 in kodo millet, 18 in barnyard millet, and 20 in little millet. The breeding methods employed to develop these varieties were germplasm selection (65%), pedigree selection (30%), and mutation breeding (5%) (Vetriventhan et al., 2020). To create variability in any crop, hybridization is a prerequisite. In crop improvement, developing a diverse population to exercise selection for desirable characters, hybridization is the preliminary step. The floral morphology and floral biology of the cross-pollinated crops ensure ease in hybridization, unlike the self-pollinated crops. Floral morphology, tiny florets, and anthesis behavior are the major hindrances to crossing in small millets. However, pedigree selection followed by hybridization to develop new varieties is a crucial breeding strategy for small millets. Manual emasculation of florets is practically very difficult in small millets; therefore, the contact method of hybridization is followed (Manjappa et al., 2019). Even for skilled hands, the success rate of developing crosses is relatively low, which restricts genetic research and yields improvement studies in small millets. Small millet-based crop improvement programs have yielded positive results in recent times. Off late, few improved cultivars have been developed, although their production potential is limited. Though there is sufficient variability in the existing germplasm collections, it has not been thoroughly exploited. By following hybridization and selection in segregating populations, it is possible to use the existing variability to develop novel cultivars. To overcome these difficulties, modified crossing methods are employed in each of the small millet crops by considering their floral morphology and behavior. The objective of this review article is (i) to discuss the floral morphology of small millet crops and (ii) to discuss the various hybridization techniques employed in small millet crops. This review shall give an overview of the crossing techniques in small millet crops and encourage the readers to identify more efficient and successful crossing techniques.

## Finger millet

Finger millet is a major food crop in the semi-arid tropics of Africa and Asia and has been an indispensable component of the dry farming system (Gupta et al., 2010). It is capable of growing under extensive types of adaptations, from sea level to hilly regions of the Himalayas, and thrives under well-drained and loamy soil types (Seetharam, 1998). It is a self-pollinated, tetraploid crop with chromosome number 2*n* = 36. Finger millet has abundant dietary fiber and calcium (358 mg/100 g) and is consumed both in processed and native form (Gopalan et al., 1989). Nutritionally, finger millet is regarded as a “super cereal” due to its richness in micronutrients and minerals (National Research Council, 1996). The grains are highly resistant to pest infestation, extending their durability during storage (Mgonja et al., 2007). This feature makes it a crop suitable for famine-prone areas of the developing world to provide food and nutritional security (Mgonja et al., 2007; Gupta et al., 2017).

### Floral morphology and anthesis in finger millet

**Figure 1:**
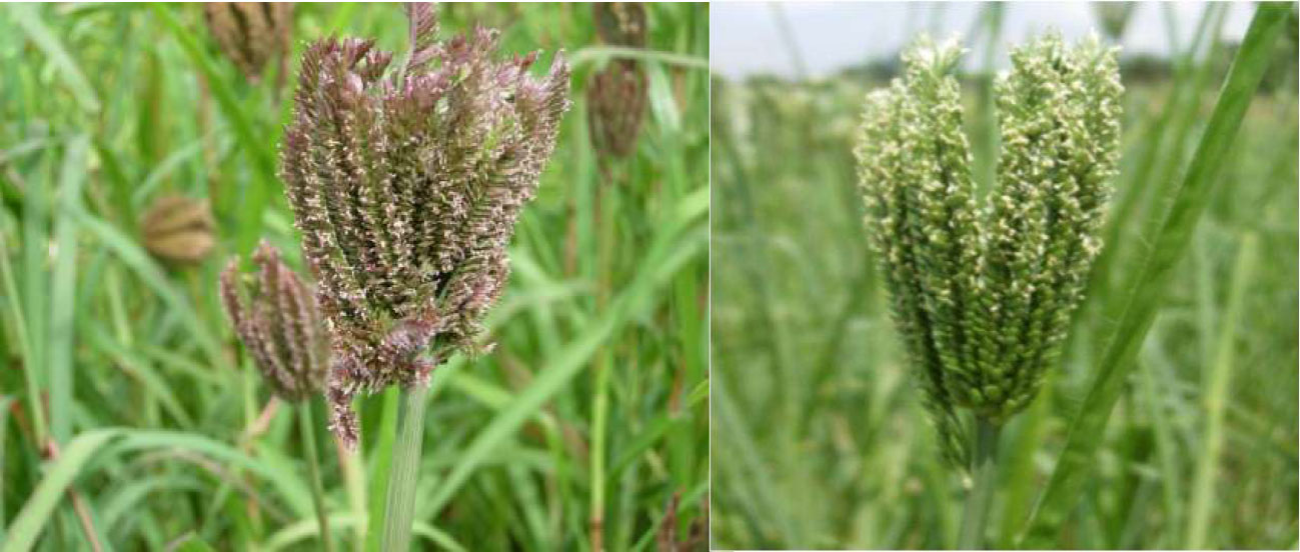
Blooming in finger millet.

**Figure 2:**
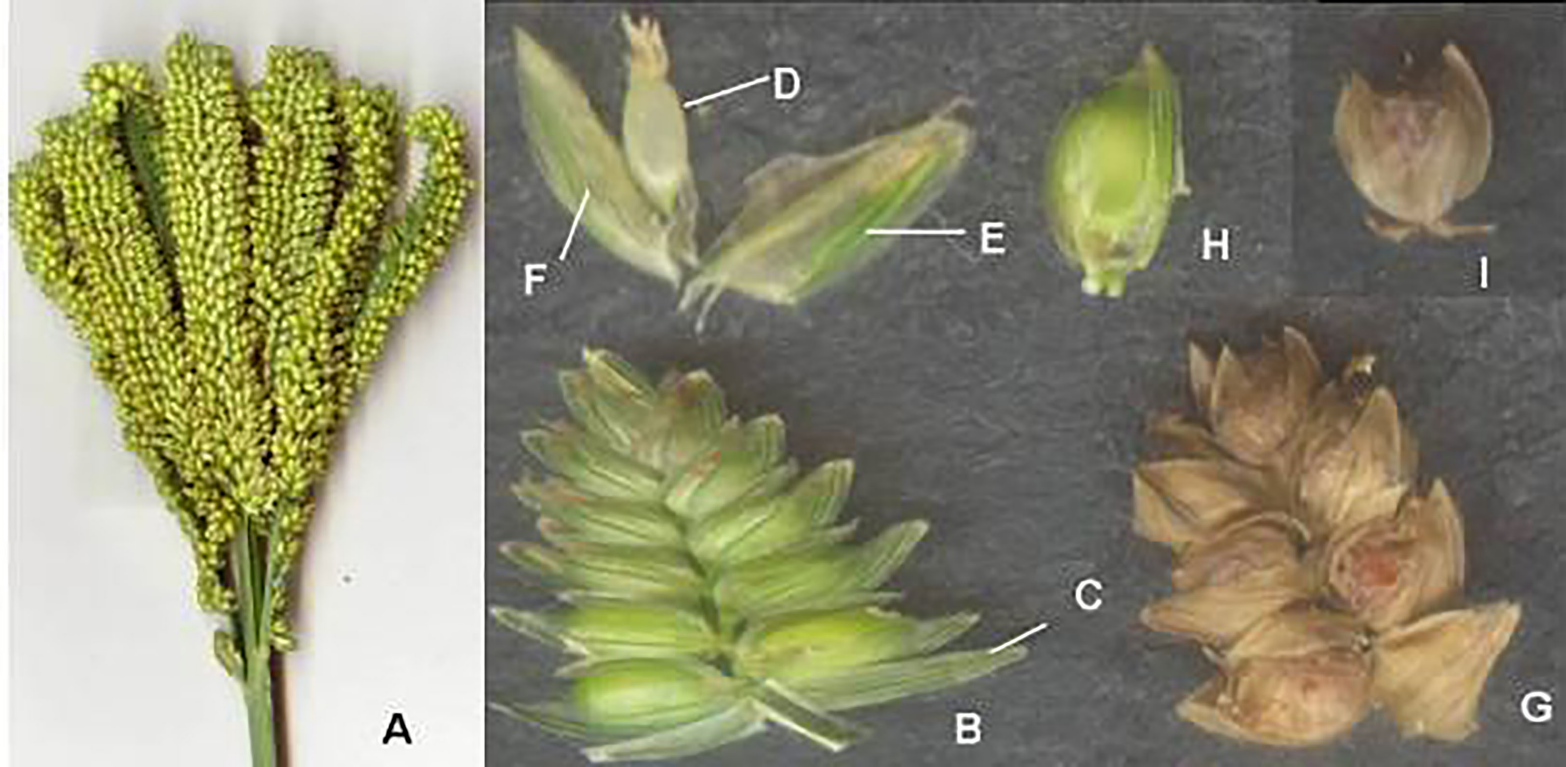
Finger millet inflorescence and its parts. **(A)** Inflorescence. **(B)** Spikelet. **(C)** Outer glume. **(D)** Ovary. **(E)** Lemma. **(F)** Palea. **(G)** Matured spikelet. **(H)** Grain within lemma and palea. **(I)** Matured grain within lemma. (Source: Gupta et al., 2011).

The inflorescence of finger millet has several fingers; each finger has in turn numerous spikelets; each spikelet further contains five florets; and each floret has three anthers around the stigma. The florets are covered by two large barren leaves, each enclosed between a pair of scales known as palea. The florets are in the axial of the lower flowering glumes known as lemma, which have tiny appendages. Near the base of the ovary, two little scaly lodicules are present (Dodake and Dhonukshe, 1998). Complete flowering takes 5 to 7 days (**Figure 1**). The gynoecium has a plumose stigma and is uni-locular and bi-carpellary with a superior ovary possessing two styles (**Figure 2**). The androecium almost completely encloses the stigma ensuring self-pollination. The filaments are relatively shorter (0.48 to 0.85 mm) than the anthers (Dodake and Dhonukshe, 1998; Emrey, 2022). Spikelet opening is basipetal. Maximum florets open on the third day of the commencement of flowering. The anthesis occurs from 1:00 to 5:00 a.m. (Gowda, 1997).

As soon as the lemma and palea separate, the stigma and anthers emerge concurrently. Anthers dehisce in a longitudinal direction before the florets emerge (Seetharam et al., 2003). At the moment of dehiscence, the anthers and sticky stigma attain the same height inside the flower. The anthers dehisce and pollinate their own stigmas. Stigma receptivity lasts for up to 5 h, while the pollen is viable for about 20 min (Dodake and Dhonukshe, 1998). Therefore, the estimated rate of natural crossover in finger millet is less than 1%.

### Hybridization techniques in finger millet

**Figure 3:**
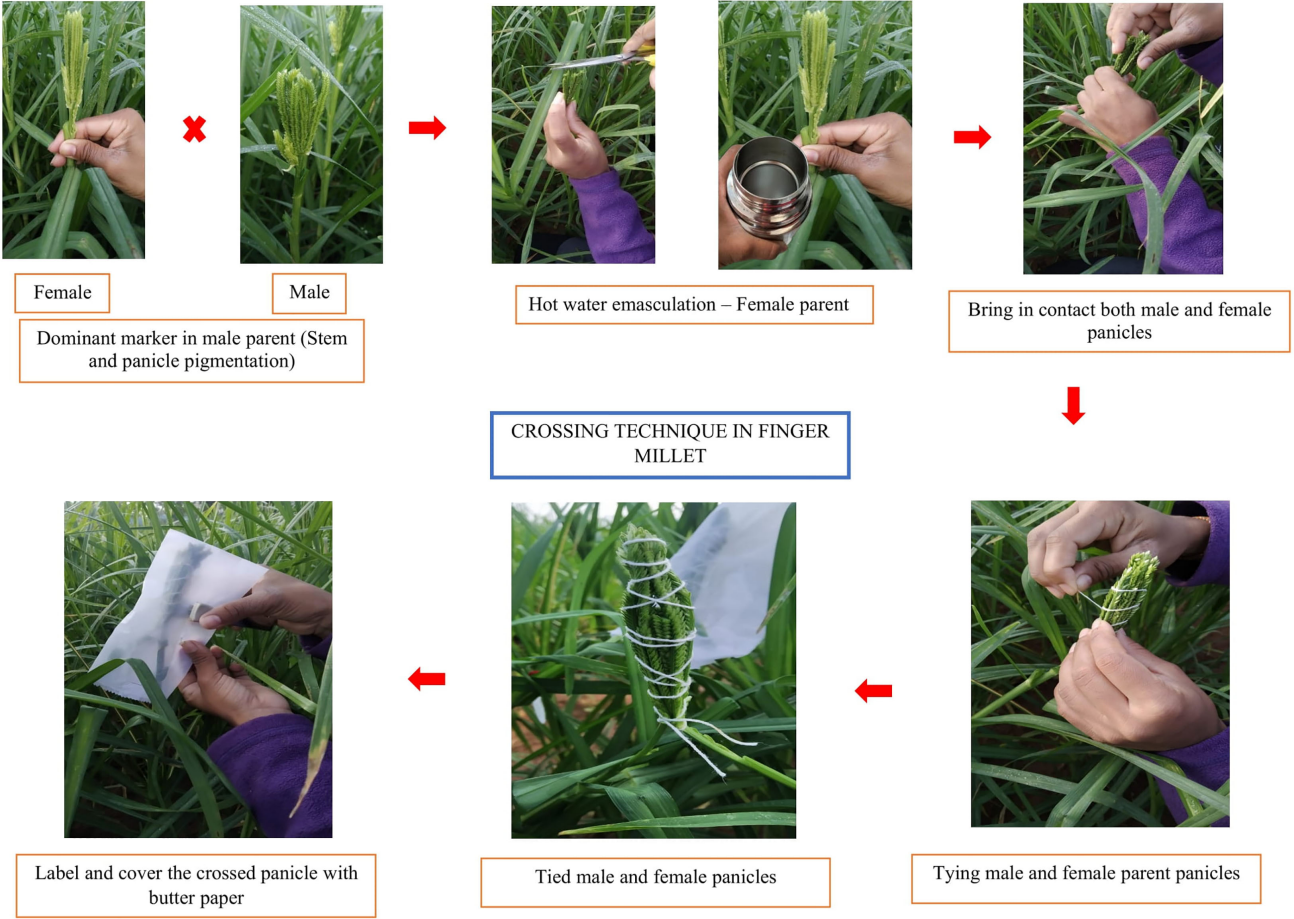
Hot water treatment method for emasculation and pollination in finger millet.

Hybridization using the contact method is the simplest and easiest (Ayyangar, 1934). In this method, the panicles at the appropriate hybridization stage are held together by intertwining the fingers of the male panicle inside the female panicle. Such panicles are covered with butter paper bags for protection and to exclude interaction with external pollen sources. Among various emasculation and pollination techniques, the contact method is widely used for hybridization and has a success rate of 2% to 3%. In the contact method for successful hybridization, genotypes with a dominant trait such as pigmentation on nodes are used as male parents (Gupta, 2006), and this helps in differentiating a crossed F_1_ from that of the selfed. A handful of crossed seeds are obtained in this method, and all the seeds from female panicles are grown to identify true hybrids. Growing all seeds to identify true hybrids requires more resources, time, space, and labor. Therefore, the hot water emasculation method is a better alternative for hybridization in finger millet (Raj et al., 1964). To overcome the disadvantages of manual removal of anthers, finger millet breeders often follow hot water treatment (Keller, 1952). In this method, the female panicles are dipped in hot water (48°C to 52°C) for 5 min. Followed by this, the panicles are air-dried and tied with a male panicle, similar to the procedure in the contact method (**Figure 3**). Reduced seeds are set in the female panicles in this procedure, and all those seeds will often be true F_1_s. This saves both time and resources to evaluate a large number of F_1_s. An increase in the temperature of hot water results in the drying up of the ears, and this is a major drawback of hot water treatment.

Temperature and humidity-induced flower openings are also reported in finger millet. In this method, the panicle is covered with a polythene bag (7.5 cm × 10 cm) lined with a moist filter at the appropriate stage, and plugged with absorbent cotton wool. The increased humidity ensures the emergence of anthers from the florets without dispersing pollen in the polythene bag. These pollens are collected and dusted on the emasculated female panicle by tapping (Nagaraja et al., 2022).

Genetic male sterility (GMS) is also reported in finger millet and is used as one of the techniques for enhancing hybridization and creating variability. Application of gibberellic acid (GA3), a gametocide, at 1,600 parts per million (ppm) at the 5th leaf stage is effective in inducing male sterility. However, this method is not utilized in hybridization programs due to maintenance problems; chemically induced male sterility needs to be induced for every season/generation, which is a resource- and time-consuming procedure and also hazardous to the people involved in it. The genotype ‘INFM 95001’ is derived from the GMS method. A partial GMS line (PS 1-IC0598201; INGR14015) in the varietal background of GPU-28 is identified, but its practical utility is narrow due to varying levels of sterility/fertility across different locations and genetic backgrounds (Gowda et al., 2014).

Numerous partial male sterile (PS1) and virescent (accession no. GE 1) mutants isolated at the Project Coordinating Unit (Small Millets), ICAR-AICRP on small millets, Bengaluru, India, are characterized. The PS1 is an ethyl methane sulphonate (EMS)-generated mutant that sets approximately 10% of its seeds upon bagging, 20% under open pollination, and up to 49% in controlled crossing. It is a monogenic recessive trait and segregates in F_2_ and F_3_ generations, signifying the existence of gametic selection. Pollen germination under a fluorescence microscope proved that disruption in both pollen germination and pollen tube growth is the cause of partial male sterility. The identification of hybrid derivatives from the pool of progeny plants can be done only after the seeds set, thus requiring more space and time, which restricts the breeder to handle the humongous number of crosses. A novel virescent mutant (GE1) developed at the same station (AICRP on small millets) was characterized for its utility in hybridization. Virescence is a chlorophyll-deficient trait controlled by a recessive gene that is expressed at the seedling stage and subsequently shows a progressive reversion to normal green color. Introgression of this virescence seedling marker with PS1 allows the identification of F_1_s at the seedling stage itself and thus enables a breeder to handle enormous crosses in less space. In order to enhance recombination breeding, 33 diverse male sterile-virescent lines were developed (Manjappa et al., 2022). Twelve partial sterile lines, five virescence lines, and seven virescences with PS lines were developed at AICRP (Small Millets), UAS, Bengaluru, India. Among these, 10 lines were crossed with released varieties and germplasm accessions for evaluation. Extensive studies on the usage of gametocides, along with the search for other stable male sterile systems and mechanisms like protogyny for effective utilization of heterosis in finger millet, are essential (Oduori and Kanyenji, 2007).

## Foxtail millet

Foxtail millet, a crop of the ancient past also known as Italian millet, is cultivated for food grain, hay, and pasture purpose. It is believed to have been domesticated in the highlands of central China (Yang et al., 2012). It is a diploid (2*n* = 18) self-pollinated crop with a cross-pollination of about 4% (Li et al., 1935). Natural outcrossing between foxtail millet and its wild progenitor *Setaria viridis* was up to 2.20% (reported up to 4.0%, if different panicles are bagged together), and interspecific hybridization occurred partly in the same order of magnitude but was different according to the distances of the male and female lines and the design of the experimental plots (Boudry et al., 1993). The countries leading in foxtail production are China, India, and Japan. In India, its cultivation is restricted to the states of Andhra Pradesh, Karnataka, Tamil Nadu, and some parts of Maharashtra. The grain has a substantial amount of protein, dietary fiber, vitamins, minerals, and beta-carotene and also contains reduced quantities of fat (Murugan and Nirmalakumari, 2006).

### Floral biology and anthesis in foxtail millet

**Figure 4:**
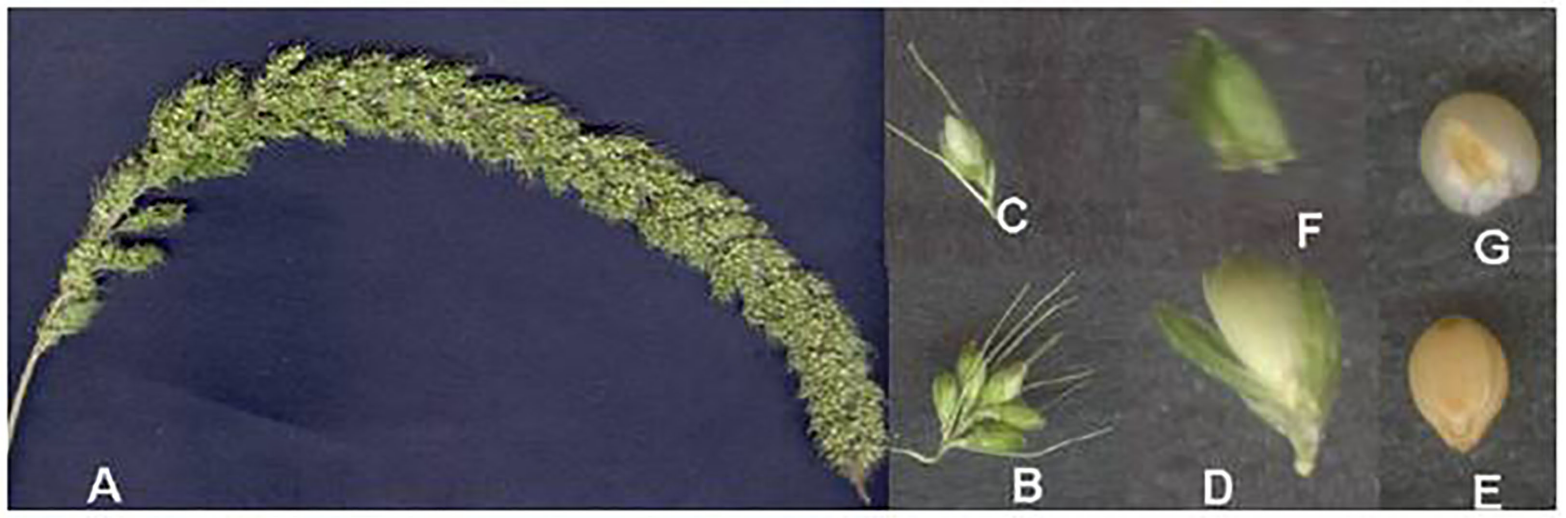
Foxtail millet inflorescence and its parts. **(A)** Inflorescence. **(B)** Spikelet’s cluster. **(C)** Subtended spikelet. **(D)** Opened spikelet. **(E)** Grain enclosed in lemma and palea. **(F)** Outer glume. **(G)** Grain. (Source: Gupta et al., 2011).

**Figure 5:**
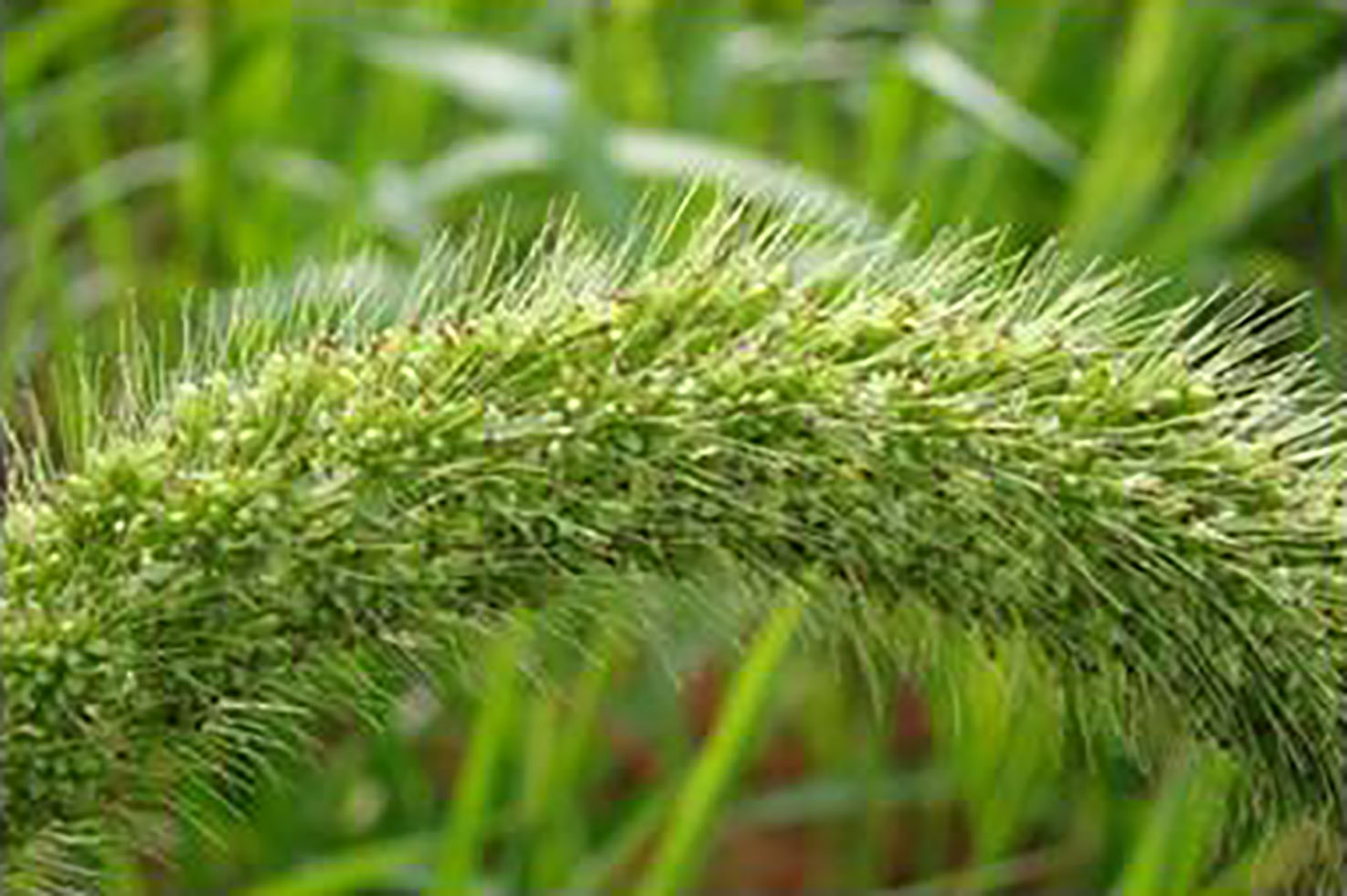
Blooming in foxtail millet.

The inflorescence, a terminal spike, has a primary stalk with side branches that are dwarf in nature and bears spikes and bristles (Siles et al., 2001). Each spikelet embodies two glumes that enfold a sterile flower in the lower position and a fertile or bisexual flower in the upper position with three stamens. An oval ovary that is long and smooth with two long styles with feathery ends is present (Siles et al., 2001; Nirmalakumari and Vetriventhan, 2010) (**Figure 4**). Anthers are white or yellow in color, and the ovary has two lodicules (Jayaraman et al., 1997). The flowering of the main spike is basipetal (Sundararaj and Thulasidas, 1976). Anthesis varies with the environment under which foxtail millet is grown, but in general, it occurs at midnight and between 8:00 and 10 a.m. (Siles et al., 2001). Complete flowering of the earhead takes 10 to 15 days (**Figure 5**). Each floret remains open for about 30 min, while complete blooming takes 80 min (Malm and Rachie, 1971). Maximum florets open on the 6th day of emergence (Sundararaj and Thulasidas, 1976). Environmental variables like humidity and temperature impact the process of pollination (Malm and Rachie, 1971). Lower temperatures and high humidity impact the rate of anthesis positively (Rangaswami et al., 1933; Heh et al., 1937).

### Hybridization techniques in foxtail millet

Owing to its self-pollinated behavior, pure line selection is widely followed. Also, hybridization followed by the pedigree method is also used to develop varieties. Sufficiently high parent heterosis for grain yield is expressed, and based on average heterosis, the F_1_s generate about 60%–70% greater average yield than parents (Siles et al., 2004). The contact method of crossing is used for hybridization, but the frequency of obtaining true hybrids is low. Of late, hybridization using the hot water emasculation method is extensively used. Generally, a dominant trait is used as a marker is used to identify true F_1_s. Rapid generational advancement and advancement with selection are widely followed (Jijau, 1989).

Siles et al. (2001) demonstrated emasculation followed by hybridization in foxtail millet. In this method, after the emergence of the first anther and before the bursting of the pollen sacs, emasculation is done in female plants. The emasculated flowers are tagged and covered with a glassine bag, whereas the untagged ones are cut off. To facilitate pollination, the emasculated female spike is placed below the male spike that has initiated pollen shedding, followed by covering it with a glassine bag. During this process, the pollen showers from male spikes to female ones, which enhances fertilization. After the third day of pollination, the male spike is separated from the female spike and discarded; simultaneously, the development of new florets is examined on the female spike. In this crossing technique, an average of 75% seed set and 90% true hybrid seeds are obtained (**Table 1**). The process of emasculation and pollination in foxtail millet is constrained due to the narrow time interval between the opening of the flower and pollen shedding. In foxtail millet, as anthers push themselves out from the floret, facilitating pollen shedding, the anthers must be cut off meticulously and briskly; hence, it is difficult to avoid contamination due to self-pollination. In addition, as pollination is done immediately after emasculation, this limits the number of crosses that can be performed per person/per day. An alternate method was reported wherein mature panicles were immersed in warm water to heat-kill developing pollen grains (Miyaji and Samura, 1954; Chang, 1958; Darmency and Pernes, 1985; Wang et al., 1998), with the advantage of performing crosses on numerous panicles. However, the temperature and duration of heat treatment varied widely across experiments (e.g., 47°C for 10 min and 42°C for 20 min). For emasculation, hot water treatment (48°C temperature) for 3 to 6 min accompanied by bagging the emasculated flowers ensures the elimination of selfed seeds resulting from newly developed flowers after emasculation. This method is also followed in the crossing of other *Setaria*.

**Table 1:** Percent seed set upon artificial hybridization in various millets.

Millet	Parents	Method	% Seed set	Location	Author
Foxtail millet	PI 458628, PI 531445, PI473598, Red Siberian, NESE 62, Golden German, and PI464223	Emasculation and pollination	75%	University of Nebraska-Lincoln	Siles et al. (2001)
Foxtail millet	–	Emasculation and pollination	37.50%–50.30%	–	Jasovskij. (1960)
Proso millet	–	Mechanical stimulation by rubbing and warming them between 0800 and 0900 prior to the natural opening	80%	Panhandle experiment station	Nelson (1984)
Proso millet and little millet	Proso millet: JK × Peddasame Little millet: GPUP 8 × K1	Cold water treatment (SMUASB) method	50%–60%	UAS, Bengaluru	Nandini et al. (2019)
Little millet	GV-1 × WV 126 GV-2 × WV 126 GNV-3 × WV 126	SMUASB method	50%–60%	AICRP-Small Millets, Hill Millet Research Station, NAU, Waghai, Gujarat, India	Patil (2021)
Finger millet	PR202 and IE 2606	Hot water treatment and hand emasculation	Hot water treatment: 52%–80% Hand emasculation: 16%–40%	ICRISAT, Hyderabad	Krishna et al. (2020)

Hui et al. (2013) used controlled environments for synchronized flowering and increased reproducibility in *S. viridis*. Crosses were performed using a transgenic foxtail millet line as a male parent containing the glucuronidase (GUS) reporter gene to facilitate the identification of successful crosses. The GUS transgene enabled the scoring of F_1_i individuals and thus provided an easy qualitative assay to determine the efficiency of emasculation and pollination.

To utilize heterosis, male sterile lines are identified in China. It is known that male sterility is governed by a single recessive gene in foxtail millet. Various male sterile methods, like photo- and thermosensitive genetic male sterility (PTGMS) (Cui et al., 1979; Wang et al., 1993; Zhao et al., 1996; Hao et al., 2009), gene interaction male sterility (Hu et al., 1986), cytoplasmic male sterility (Zhu et al., 1991), and cytoplasmic genetic male sterility (Zhi et al., 2007), were used to identify male sterile lines in foxtail millet. Genetic male sterility has been exploited in foxtail millet to develop hybrid cultivars, namely Jigu 16 and Suanxi 28 × Zhangnong 10 (Cui et al., 1979; Du and Wang, 1997). The first foxtail millet PTGMS line ‘292’ (Cai 5 × Ce35-1) was made male fertile to some degree by a short photoperiod treatment (13 h or shorter) and made male sterile by a longer photoperiod treatment (14 h or longer) at the Baxia Institute of Agricultural Sciences (Zhao et al., 1996). Nugroho et al. (2020) provided evidence that warm water treatment is recommended to induce male sterility in foxtail millet as it is effective in killing pollen, inflicting unproductive rachis in the upper panicle and pollination zones, and keeping the stigma receptive. So we can say that more efforts are needed to exploit male sterility in order to facilitate the development of hybrid progenies so that new segregants could be generated and genetic recombinations could be enhanced in foxtail millet breeding schemes.

## Proso millet

Proso millet is a diploid (2*n* = 36), short-season crop with the ability to tolerate increasing temperatures and water-deprived conditions (Changmei and Dorothy, 2014). It is a self-pollinated crop; nevertheless, natural cross-pollination exceeding >10% is also noticed (Popov, 1946). This C_4_ crop is cultivated across India, Nepal, Western Burma, Pakistan, Sri Lanka, and South East Asian countries. Whereas in India, it is cultivated across the states of Tamil Nadu, Karnataka, Andhra Pradesh, and Uttarakhand, accounting for more than half a million-hectare area. In addition to being used as food, it is majorly utilized as a feed for birds and animals in various parts of Asia (Rajput et al., 2014). Nutritionally, the grains are akin or superior to cereals, and the grain protein is composed of essential amino acids (Gomashe, 2017).

### Floral biology and anthesis in proso millet

**Figure 6:**
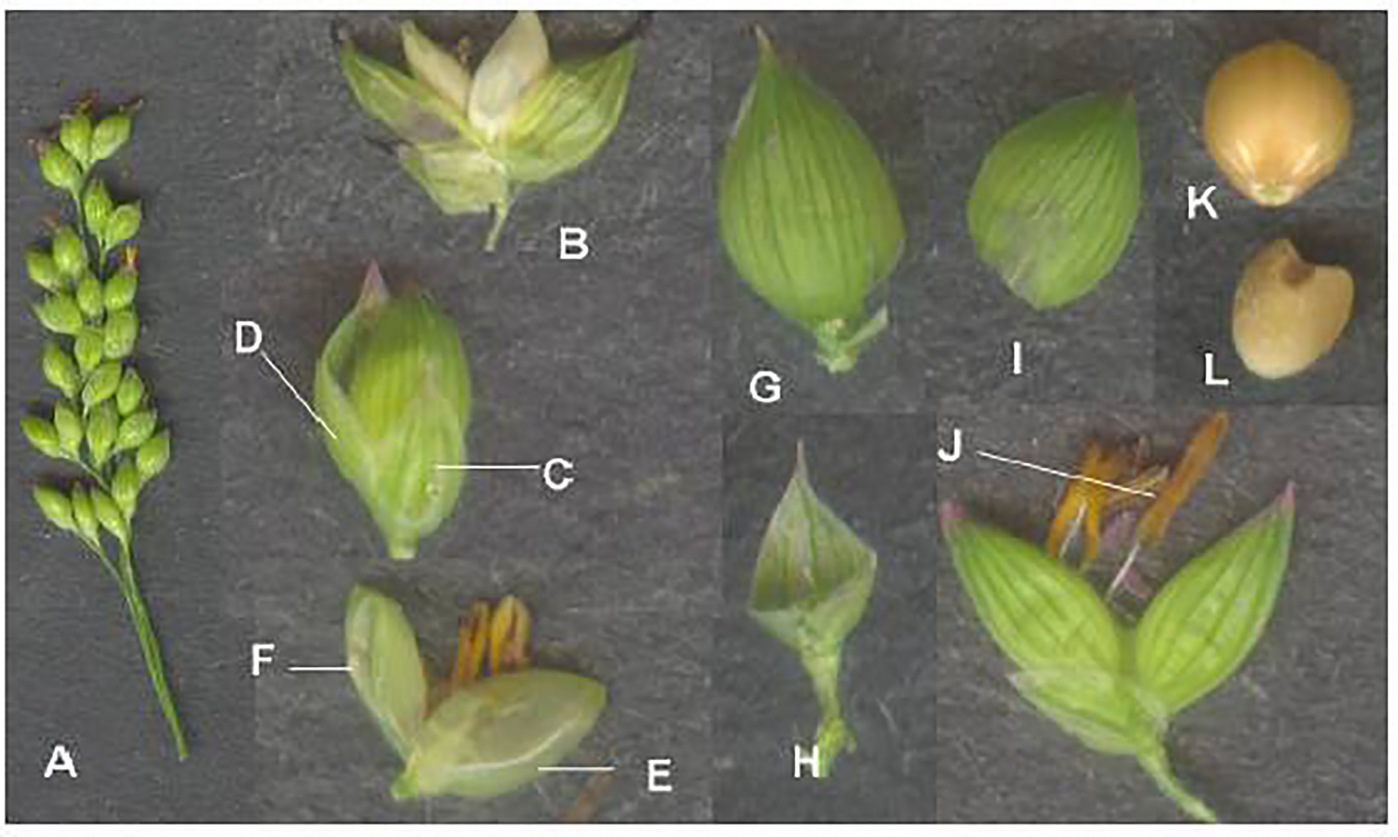
Prosomillet inflorescence and its parts. **(A)** Inflorescence. **(B)** Opened spikelet. **(C)** Outer glume. **(D)** Inner glume. **(E)** Inner lemma; **(F)** Palea; **(G)**Inner glume; **(H)**Outer glume; **(I)** Upper lemma. **(J)** Anther. **(K)** Grain enclosed in lemma and palea. **(L)** Grain. (Source: Gupta et al., 2011).

**Figure 7:**
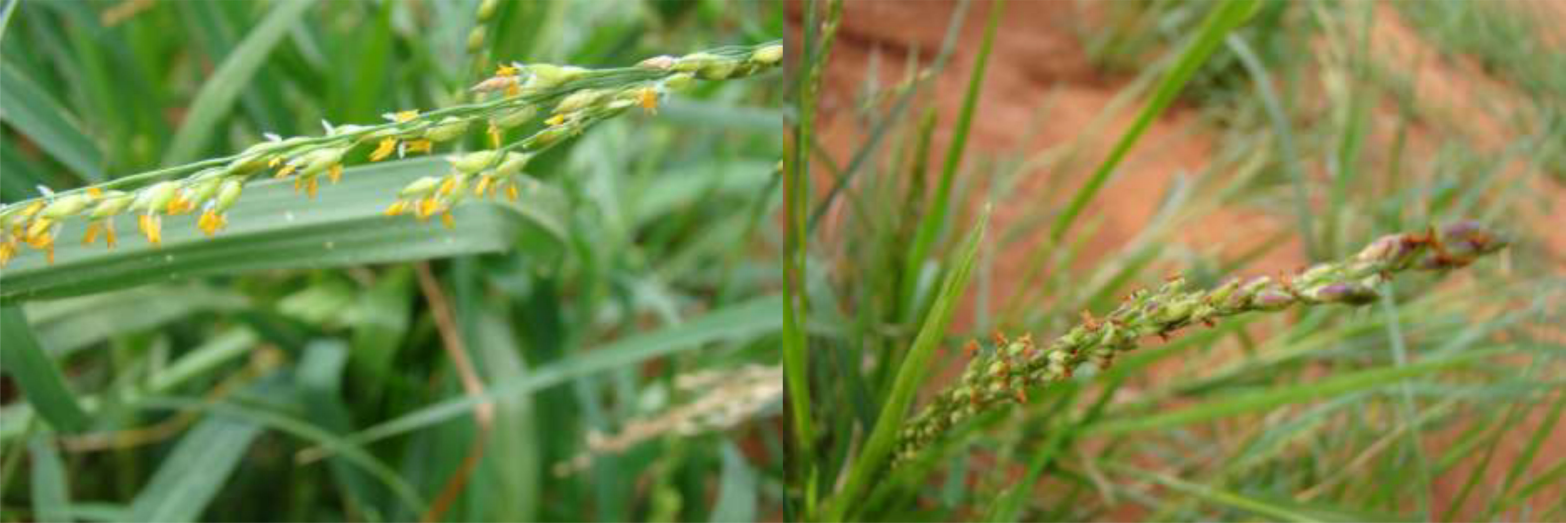
Blooming in proso millet.

The inflorescence may be an open or compact type that bears numerous spikelets and is droopy (Hulse et al., 1980). Each spikelet has two glumes and lemmas. The outer and inner glumes are dwarf and long, respectively. A single floret is present in a lemma. A long, sterile, and stamen-less floret is present in the lower lemma, while a short and fertile one is in the upper lemma. The lower palea is reduced, and the upper lemma is prominent (Seetharam et al., 2003) (**Figure 6**). Three stamens are present, which are tan or amber or blackish or dark brown in color. The style is bifid, and the stigma is plumose (Nanda and Agrawal, 2008). Flowering is in the basipetal direction (Sundararaj and Thulasidas, 1976). Anthesis initiates from 10:00 a.m. to 12:00 p.m. (Jayaraman et al., 1997). The complete process of anthesis takes 12–15 days (**Figure 7**). The stigma receptivity and pollen shedding occur concurrently. Anthers are sticky and do not shed pollen, while the florets are closed (Nelson, 1984). Immediately after the floret opening, the anthers dry out and initiate pollen shedding. The florets are opened for about 10–15 min (Zhuravel, 1956). The environmental factors, *viz*., optimal temperature, moderate humidity, and blazing sunshine, stimulate the process of flowering. It is observed that on dull and murky days, flowering is hampered; hence, panicles are heated with the aid of a lens to stimulate flowering. Flowering can be stimulated by heating the panicle with a lens, with a maximum response between 10:00 and 11:00 a.m. (Belov, 1916). Several methods of emasculating proso millet have been proposed, including hot air (Vasilev, 1966), hot water (Primak and Jakovlev, 1964), rubbing the panicles and dipping them in water (Jasovskij, 1960), and emasculation followed by bagging for 2 days prior to pollination. Several workers (Zhuravel, 1956; Ilin, 1960; Jasovskij, 1960) proposed pollination immediately after emasculation.

## Little millet

Little millet, one of the coarse cereals consumed as rice, is a self-pollinated, and tetraploid crop with chromosome number 2*n* = 36. It is grown across India, China, Nepal, Sri Lanka, and Western Burma. In India, it is cultivated in Karnataka, Tamil Nadu, Bihar, Andhra Pradesh, Maharashtra, Madhya Pradesh, Orissa, Uttar Pradesh, and Gujarat states. It is recognized for its resistance to drought, owing to which it is one of the least water-demanding crops and can be grown in both subtropical and tropical climates (Doggett, 1989). It is composed of protein, vitamins, carbohydrates, and minerals. A balanced amino acid profile is seen in proteins, besides being a good source of methionine, lysine, and cystine as well.

### Floral biology and anthesis in little millet

**Figure 8:**
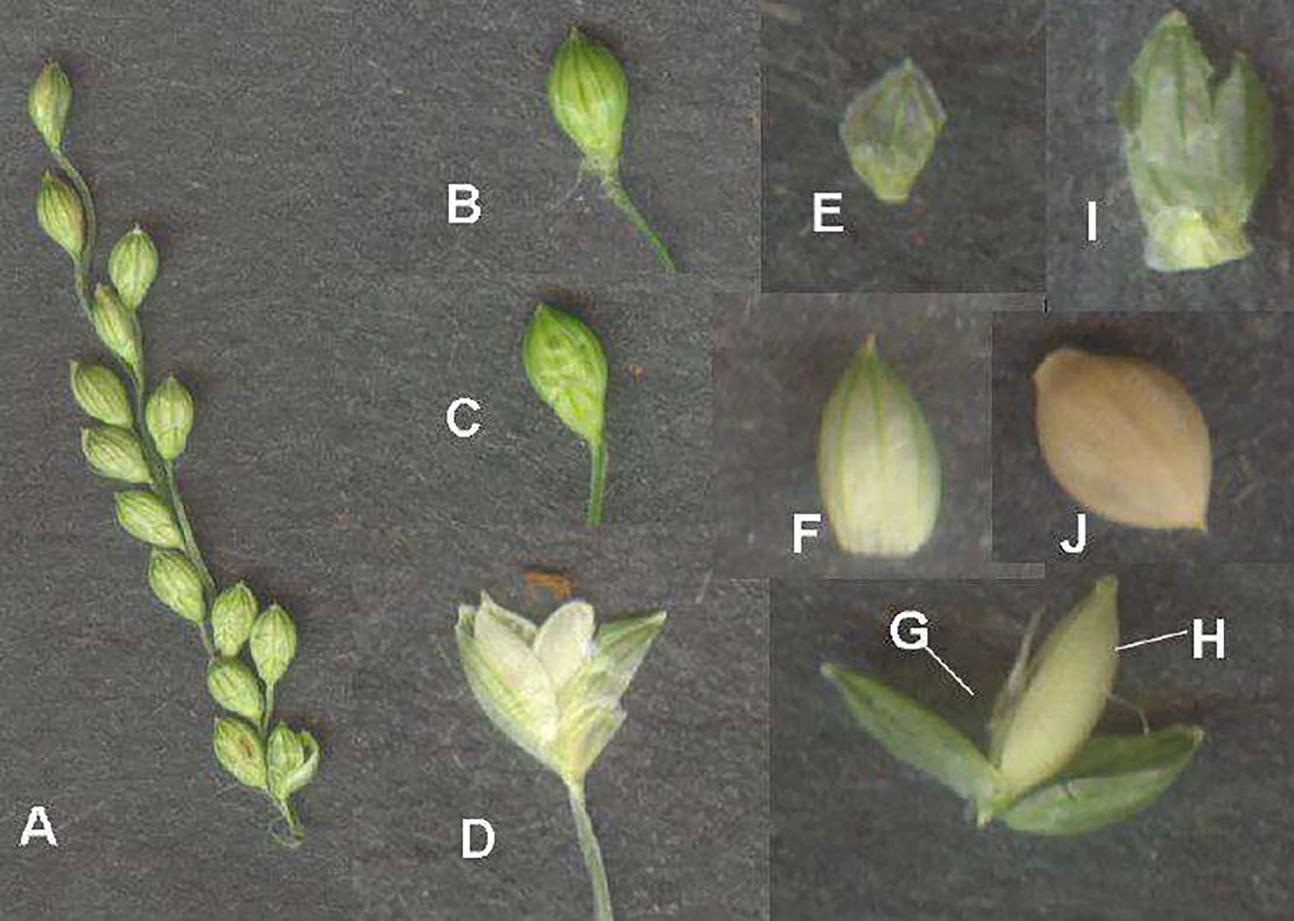
Little millet inflorescence and its parts. **(A)** Inflorescence. **(B)** Spikelet. **(C)** Abaxial view of spikelet. **(D)** Opened spikelet. **(E)** Outer glume. **(F)** First lemma. **(G)** Sterile floret. **(H)** Fertile floret. **(I)** Upper glume. **(J)** Grain enclosed in lemma and palea. (Source: Gupta et al., 2011).

The inflorescence of little millet is a panicle that is 15 to 45 cm long, 1 to 5 cm broad, and constricted or thyrsiform (Seetharam et al., 2003). The persistent spikelet is 2 to 3.5 mm in length (Bor, 1960). When panicles reach maturity, they become scabrous and droop. Each spikelet flowers for around 2 to 5 min. The top one is fertile or bisexual without rachilla expansion, whereas the lower one is sterile. The fertile flower is enclosed by lemma II and its palea, while the staminated or sterile flower is enclosed by lemma I and its palea (Sundararaj and Thulasidas, 1976). Spikelets are elliptical, dorsally compressed, and acute, with three anthers of about 1.5 mm in length. The glume that reaches the apex of florets is thinner than the fertile lemma (**Figure 8**). The lower glume is ovate, membranous, with no keels, possesses one to three veins, and is about 0.7 to 1.2 mm long. The apex of the lower glume is acute. An ovate upper glume is larger than the lower glume, and the keel is absent. It has 11 to 15 veins (Nanda and Agrawal, 2008).

**Figure 9:**
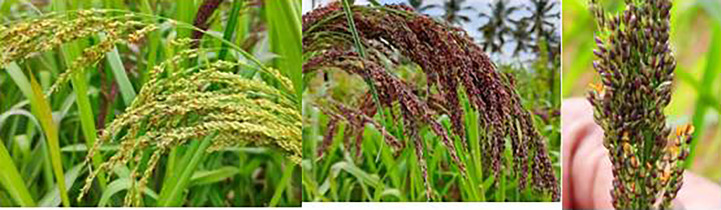
Blooming in little millet.

The spikelet opening begins on the second or third day after the appearance of the panicle. The process of flowering is basipetal. Maximum flower opening is seen on the sixth or seventh day of flowering. For complete flowering in a panicle, about 2 weeks are required (Sundararaj and Thulasidas, 1976). The panicle blossoms around 9:30 to 10:30 a.m. (Jayaraman et al., 1997) (**Figure 9**). Since the glumes are opened for a limited duration, self-pollination is the rule (Seetharam et al., 2003). It takes 2 to 5 min to complete the process of anthesis.

### Hybridization techniques in little millet and proso millet

These crops frequently use the contact method of hybridization, which involves planting a desired male parent next to a female parent. Male and female panicles are tied loosely. After pollination and fertilization are completed, both are separated. While choosing a male parent, it should have morphological markers to aid in the identification of true *F*_1_s (http://agritech.tnau.ac.in). The success rate of attaining true F_1_s is quite low at about 2% to 3%. To identify true F_1_s, a large plant population must be raised. More space and resources are required for evaluation (Nandini et al., 2019).

For crossing, hand emasculation followed by pollination is also followed. Anthers and stamens of the flowers that are about to open the next day are cut off without disturbing the female reproductive organs. Often, this is done in the evenings, and pollination is done in the following morning. Male flowers that are about to dehisce the following day are brought into contact with the emasculated female flower, followed by which they are tied and covered with a butter paper bag to facilitate pollination. It takes 2 to 5 days for natural cross-pollination to complete. To identify true hybrids, reliable genetic markers are used (http://agritech.tnau.ac.in). The major disadvantage of this procedure is that the lemma and palea are extremely tight, making the flower difficult to open before regular anthesis without damaging it, thus preventing the development of seeds. Moreover, the timespan required for the anthesis of the first floret on a panicle and that of the last floret is another drawback. This makes it challenging to decide when to emasculate before anthesis (Jasovskij, 1960).

To subjugate this issue, Nelson (1984) developed a method to choose flowers to be emasculated in proso millet. In this technique, those panicles whose florets are already opened are selected. Such panicles are rubbed against the palm of our hands to enhance the opening of the remaining unopened florets, and further water (room temperature) is sprayed to prevent another dehiscence and to keep them moist. All opened florets are emasculated. The florets that are not emasculated are clipped off, which are the ones at the top that have been fertilized previously and the immature florets at the bottom. The ideal time to emasculate is around 8:00 to 9:00 a.m. During this time, effective emasculation of the florets is possible. After 15 min of emasculation, fertilization is done. The panicle of the male parent is rubbed to open the florets; such florets are placed in a glassine bag and inverted over the emasculated panicle to facilitate pollination. The crossed panicles are left undisturbed for 5 days to ensure the preservation of moisture. The advantage of this method is that lemma and palea open naturally rather than being forced to do so. While the disadvantage is that stigma injury may occur during emasculation, resulting in no seed set.

As an alternative to the worrisome manual removal of anthers, the hot water method of emasculation is employed (Keller, 1952). In this method, panicles that are expected to open in the next 2 to 3 days are dipped in hot water (52°C) for 2 min. Srivastava and Yadav (1972) noticed that the panicles of little millet were successfully emasculated when dipped in hot water (49°C) for 8 to 10 min. Similar results were reported by Primak and Jakovlev (1964) in proso millet, who found that hot water treatment (50°C) for 5 min improved seed set. Once emasculation is done, the panicles of the male parent that are expected to open the succeeding day are held tightly to the emasculated panicle of the female parent and covered with a butter paper bag to ensure pollination. The need for an appropriate apparatus that maintains the required optimum water temperature is a con of this method. Since the stigma is affected by temperature, this may result in a reduced quantity of seed sets.

**Figure 10:**
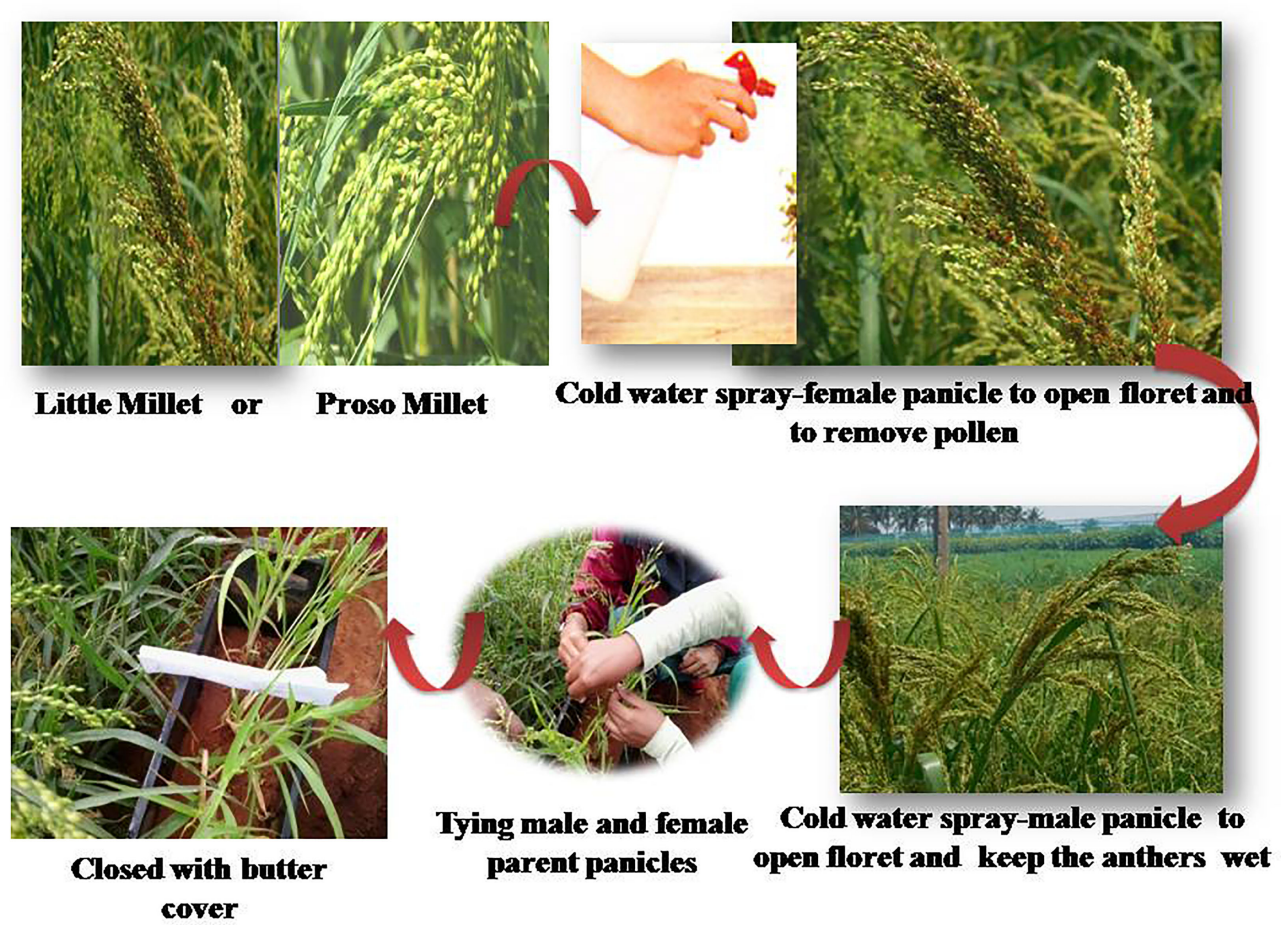
Modified crossing technique (SMUASB) followed in proso millet and little millet. (Source: Nandini et al., 2019).

Seetharam et al. (1986) proposed the Union of Soviet Socialist Republics (USSR) modify the crossing method to repress the issues of hand emasculation and hot water treatment. In this approach, florets are gently massaged with a palm, ensuring mechanical stimulation, after which the florets open within 2 to 3 min earlier than normal flower opening. Such florets are immersed in water at ambient temperature to avoid the bursting of anthers. Anthers from opened florets are removed, and the unopened florets are clipped-off. The emasculated female spike is positioned below the pollen-shedding male spike and covered with a glassine bag to promote pollination. During this process, the male spike showers pollen on the female spike, ensuring fertilization. During the anthesis period, these spikelets are shaken for two consecutive days. On the third day of pollination, the male spike is cautiously detached, and the female spike is examined for the presence of any florets that might have developed due to fertilization. This ensures the identification of crossed seeds with that of the selfed ones. Furthermore, the female spike is re-bagged until maturity, after which the crossed seeds are harvested. The pitfall in this procedure is that while massaging the panicles to mechanically stimulate floret opening and removal of anthers with the forefinger, there are chances of stigma damage (Nandini et al., 2019). The Project Coordinating Unit (Small Millets) at the University of Agricultural Sciences, Bengaluru, identified a modified crossing method (SMUASB method) in 2014 for proso millet and little millet (**Figure 10**). Due to the compactness of lemma and palea, any attempt to open florets prior to natural anthesis results in damaging the flower that results in no seed set in these two millets. Hence, the use of cold water to stimulate the mechanical opening of florets is followed in the SMUASB method.

The flower opens in the basipetal direction in both proso and little millet. For ease of crossing, alternate rows of male and female plants are grown. The most ideal time for crossing is from 8:00 to 9:00 a.m. The panicles of female plants whose first floret is already opened are sprayed with cold water (5°C to 8°C) to hasten the natural opening of florets. Furthermore, dip or wash such panicles with cold water to remove the anthers ensuring emasculation. Immature florets at the bottom of the panicle and previous self-fertilized florets at the top are clipped-off. The emasculated female panicles are pollinated by attaching them to the panicles of the male parent whose first floret is opened (by dipping or spraying cold water). Care is taken to aerate them properly. Again, water is sprayed on the tied panicles to ensure moisture in the stigma and anthers. Followed by this, a butter paper bag is covered over them to avoid contamination with undesirable pollen sources and tagged for easy identification and seeds are collected from crossed panicles. Patil (2021) recovered 50 to 60% of crossed seeds by following the SMUASB method in the cross GV 1 x WV 126, GV 2 x WV 126, and GNV-3 x WV 126 varieties of little millet (**Table 1**).

## Barnyard millet

Barnyard millet, a hexaploid (2*n* = 54) is a self-pollinated, dual-purpose crop popularly consumed as food by humans and fodder by animals. Among all the millets, barnyard millet develops at a faster pace and is harvested in a short span of 75 to 80 days. It is grown in India, Japan, China, and Korea (Upadhyaya et al., 2014), while in India, it is cultivated in the states of Uttarakhand, Madhya Pradesh, Tamil Nadu, Andhra Pradesh, Maharashtra, Karnataka, and Bihar. Barnyard millet is preferred due to its short growth period, superior performance in areas with limited water supply (Dwivedi et al., 2012), and superlative nutrient content (Saleh et al., 2013). Moreover, its grains are rich in carbohydrates (65%), protein (11%), crude fiber (13.6%), and fat (3.9%) (Hadimani and Malleshi, 1993). They also contain an abundant quantity of iron (Fe), zinc (Zn), and antioxidant compounds.

### Floral biology and anthesis in barnyard millet

**Figure 11:**
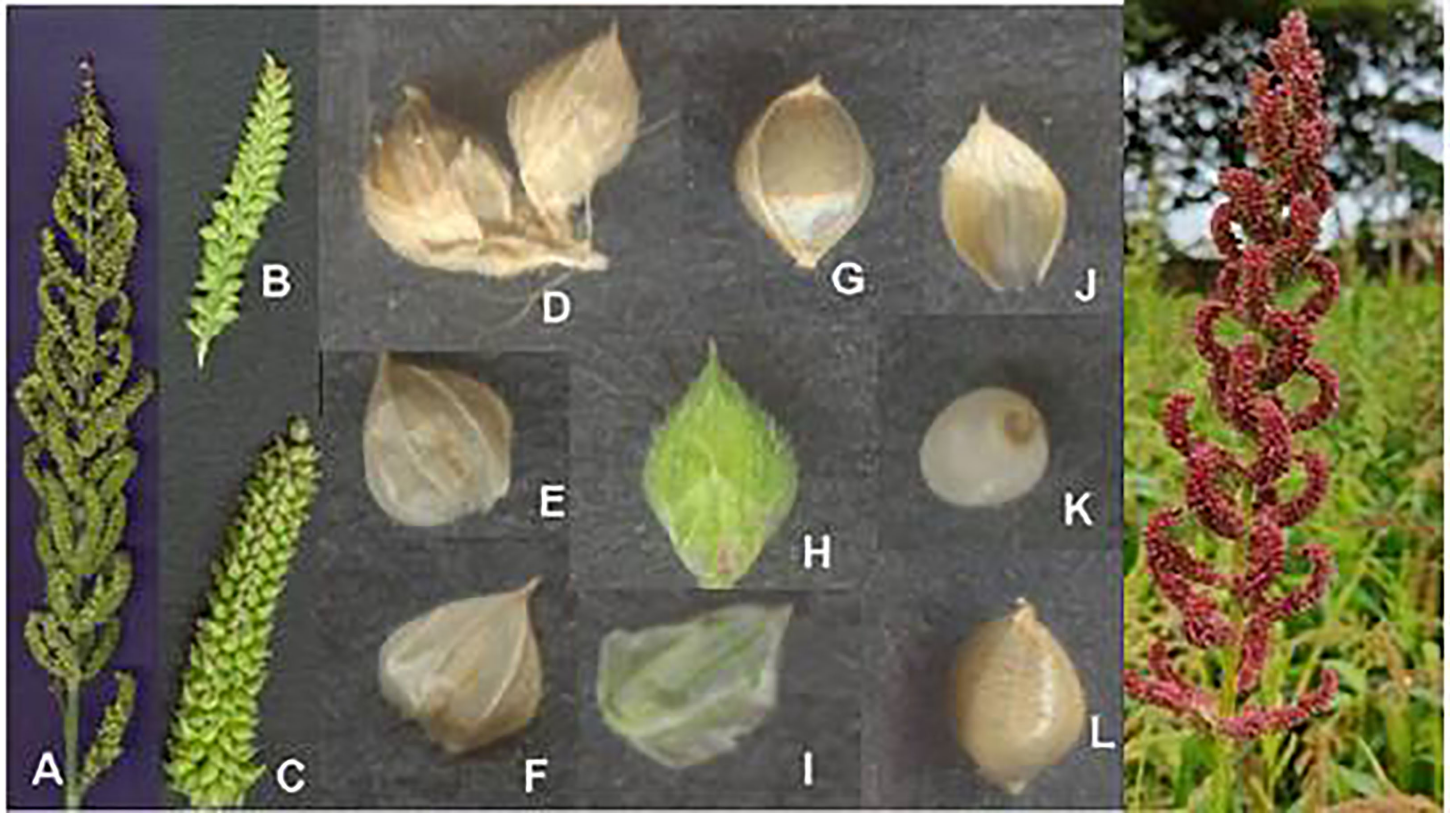
Barnyard millet inflorescence and its parts. **(A)** Inflorescence. **(B)** Arrangement of the spikelet in a raceme. **(C)** Raceme. **(D)** Spikelet. **(E)** Lower lemma. **(F)** Upper glume. **(G)** Abaxial view of fertile lemma enclosing grain. **(H)** View of spikelet from lower glume. **(I)** Lower glume. **(J)** Fertile lemma. **(K)** Grain. **(L)** Grain enclosed in lemma and palea. (Source: Gupta et al., 2011).

The inflorescence is an erect terminal panicle and rarely droops. The racemes are zero to quite a few in number and thickly congested at the apex. The spikelet is arranged in four irregular rows on triquetrous rachis. The spikes are two-flowered and ovate to an elliptical shape. The lower lemma is awnless and pointed sharply; it is also subsessile and placed on the short, rough pedicels subtended by two glumes (De Wet et al., 1983). The upper glume is shorter than the lower; usually, the lower glume is one-third of the spikelet. The pubescence is observed on the glumes and lower lemmas. A bisexual upper floret and sterile lower floret with a lemma and narrow palea are noticed (Gupta et al., 2010) (**Figure 11**). The sterile lemma contains five veins. Egg-shaped fertile lemma is plano-convex, bright and glossy, cuspidate or steeply pointed, and the margins are rolled over the palea, with the apex of the palea not being enclosed. The surface texture of the fertile lemma and palea is alike, and the palea is flat (Napper, 1965). The lemma and palea tightly enclose the grain. Three stamens, a superior ovary with two well-defined styles, and a plumose stigma are present (Sundararaj and Thulasidas, 1976).

**Figure 12:**
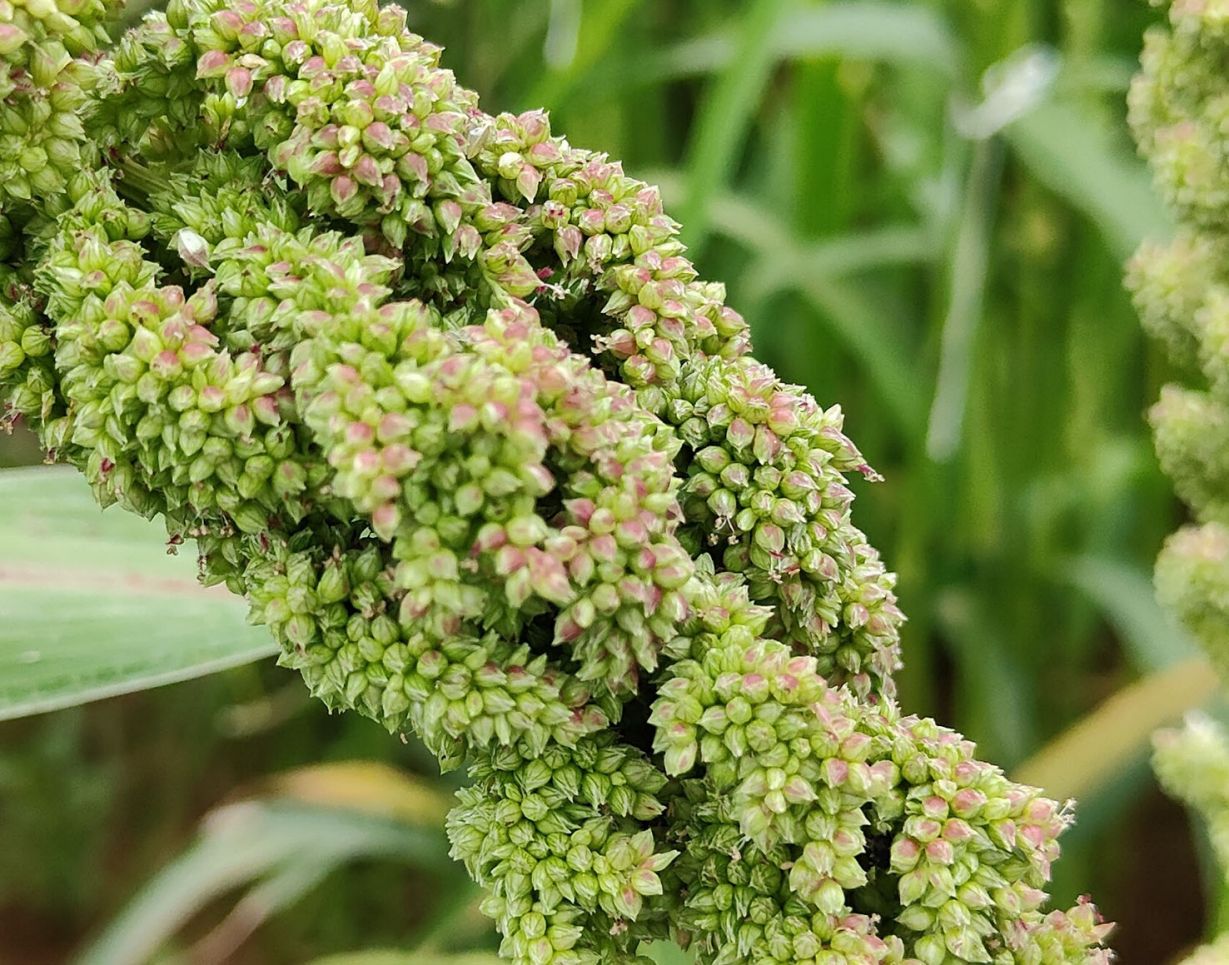
Blooming in barnyard millet.

Basipetal flowering is observed in barnyard millet. Panicle emergence takes 10 to 14 days, while complete flowering takes 10 to 15 days. A maximum number of florets open in the initial 6 to 8 days of flowering (Sundararaj and Thulasidas, 1976) (**Figure 12**). Maximum flowering is seen from 6:00 to 7:00 a.m.; generally, the process continues from 5:00 to 10:00 a.m. (Sundararaj and Thulasidas, 1976; Jayaraman et al., 1997). In a single raceme, blooming initiates from both the marginal ends and then proceeds to the center. The branches of the stigma are spread open before anther dehiscence, and the flower shuts in half an hour (Seetharam et al., 2003).

### Hybridization techniques in barnyard millet

Regularly, the contact method of crossing is followed. In addition to this, hot water treatment (48°C) for 4 to 5 min is also reported to be effective (Gupta et al., 2011). The AICRP (small millets) at Almora in Uttarakhand state of India is a pioneer in barnyard millet research and development activities. Efforts to develop and identify male sterile lines in barnyard millet are majorly initiated in this research center.

## Kodo millet

Kodo millet is native to India and was domesticated about 3,000 years ago (House et al., 1995). It is largely dispersed in moist environments across the old-world tropics. In India, it is cultivated for food grain purposes in the states of Kerala, Tamil Nadu, Rajasthan, Uttar Pradesh, and West Bengal. It is known for its excessive free radical quenching capacity, suggestive of its antioxidant property (Hegde and Chandra, 2005).

### Floral morphology and anthesis in kodo millet

**Figure 13:**
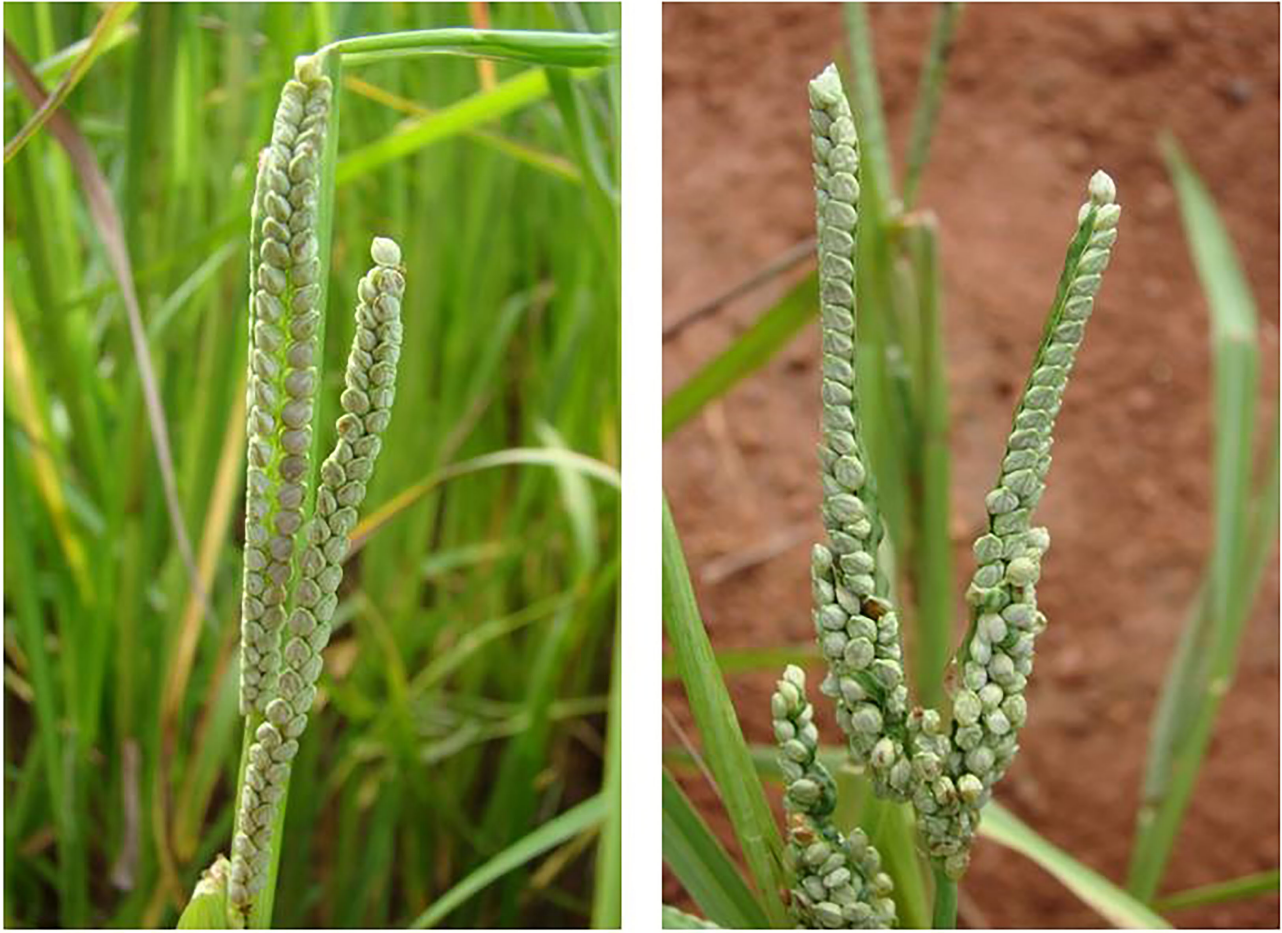
Kodo millet Panicles. **(A)** Regular type. **(B)** Irregular type.

**Figure 14:**
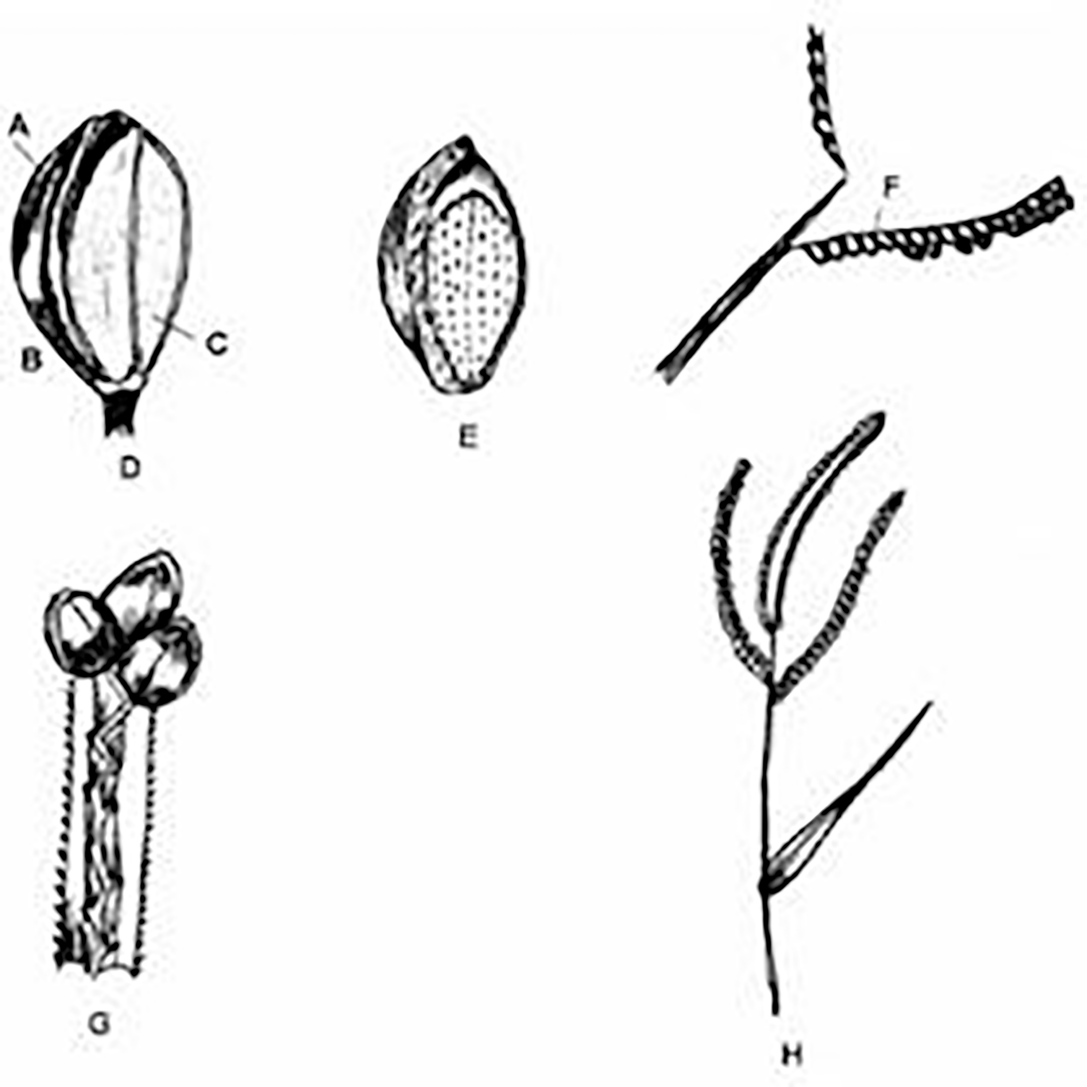
Kodo millet inflorescence and its parts. **(A)** Upper floret. **(B)** Second glume. **(C)** Lemma of lower floret. **(D)** Spikelet. **(E)** Floret. **(F)** Rachis. **(G)** Arrangement of rachis in a spikelet. **(H)** Inflorescence. (Source: Nanda and Agrawal, 2008).

The inflorescence has two to six racemes that spread widely on a sub-digitate or a short axis (Seetharam et al., 2003). Sessile spikelets are present. On a flattened rachis, the spikelets are arranged in two rows (Ramakrishna et al., 2002). Occasionally, some spikelets are paired in the middle of the raceme. Kodo millet has two types of spikelet arrangement, i.e., regular and irregular types (**Figure 13**). An alternative arrangement of spikelets in two series, i.e., long and short pedicelled, is observed (Nanda and Agrawal, 2008). One glume is missing, and the other glume is as much as that of the length of the spikelet. One lemma is similar to that of the glume whereas, the other lemma embodies two florets. The lower floret is sterile and reduced to half. The upper floret is a hermaphrodite (Sundararaj and Thulasidas, 1976). Hard, horny, and persistent husk encloses the grain (Seetharam et al., 2003) (**Figure 14**).

The flower opening in kodo millet is cleistogamous (Yadava, 1997). About 15% to 20% of flower opening is noticed, making it a self-pollinated crop. Spikelet opening initiates between 2:30 and 6:00 a.m. (Jayaraman et al., 1997). The presence of a very intact lemma makes it more difficult to artificially open the flower, which results in damaging it.

Due to the cleistogamous flowering pattern, recombination breeding is limited. Nevertheless, quite a few improved varieties have been released for commercial cultivation purposes. These are majorly developed as selections from landraces or superior germplasm accessions. Popular kodo millet varieties GK 2, APK 1, and KMV 20 are the outcome of selections from germplasm accessions. To a great degree, pureline selection is followed to develop superior-yielding cultivars. In addition, induced mutagenesis is also practiced to a great extent to generate variability in kodo millet (Yadava, 1997). The variety JK 76 was gamma-irradiated with a 5-Kr dose to develop a protogynous mutant with two rows of spikelets on the rachis; such successful endeavors in millet breeding research shall aid in recombination breeding to identify superior varieties.

## Browntop millet

Browntop millet is a drought-tolerant, climate-resilient crop that contains abundant quantities of carbohydrates, protein, dietary fiber, minerals, and vitamins. Since it can be grown in varied soil types, it is highly fit for different cropping systems. It is tetraploid at the genetic level and has a basic chromosome number of four (Basappa et al., 1987). The crop has its origins in South East Asia, and currently, it is cultivated across Western Asia, China, and Australia (Clayton et al., 2006). In India, its cultivation is confined largely to south Indian states like Karnataka, Andhra Pradesh, and Tamil Nadu (Roopa et al., 2016; Sujata et al., 2018).

### Floral biology, anthesis, and hybridization techniques in browntop millet

**Figure 15:**
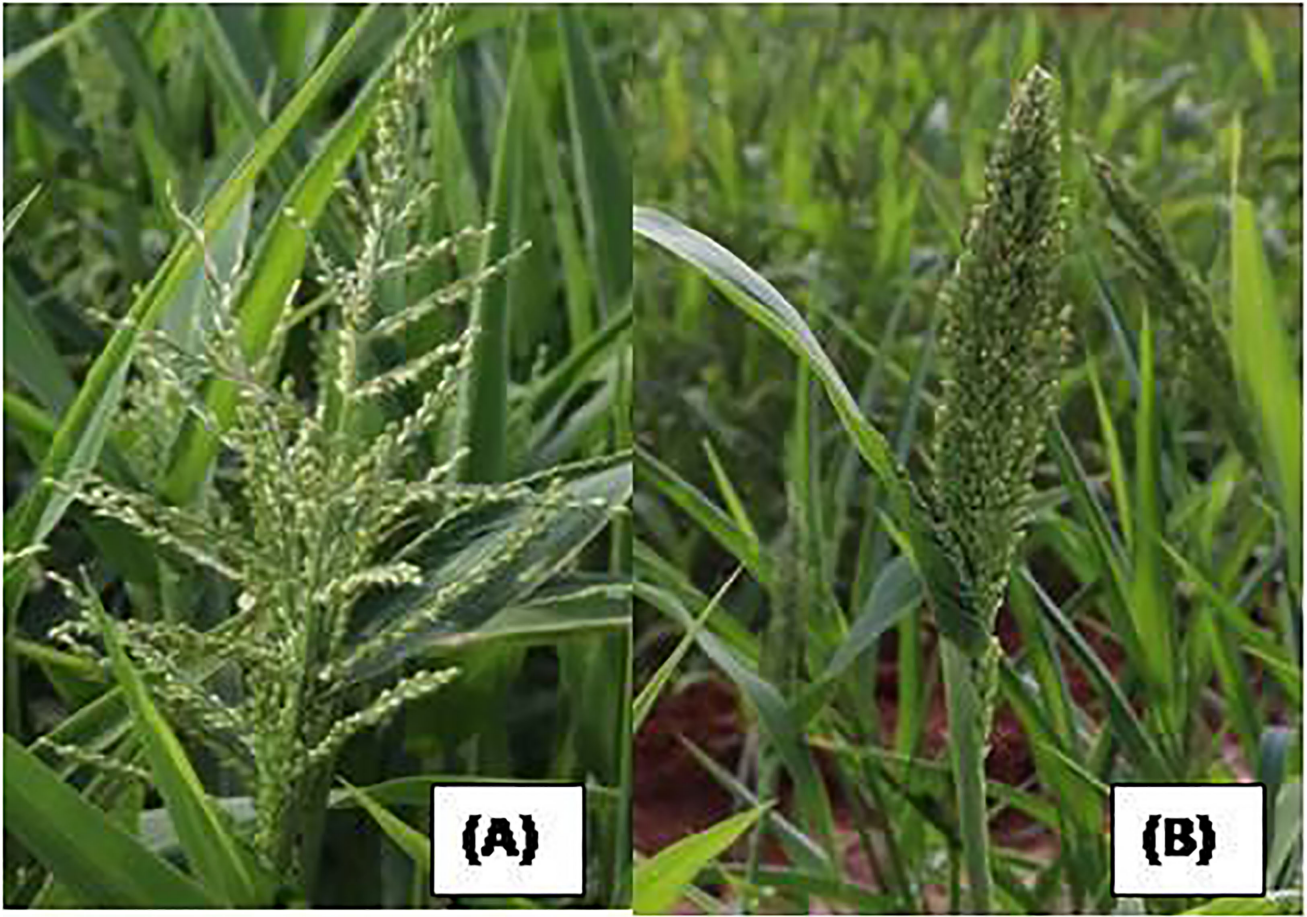
Type of panicle in browntop millet. **(A)** Open. **(B)** Close.

**Figure 16:**
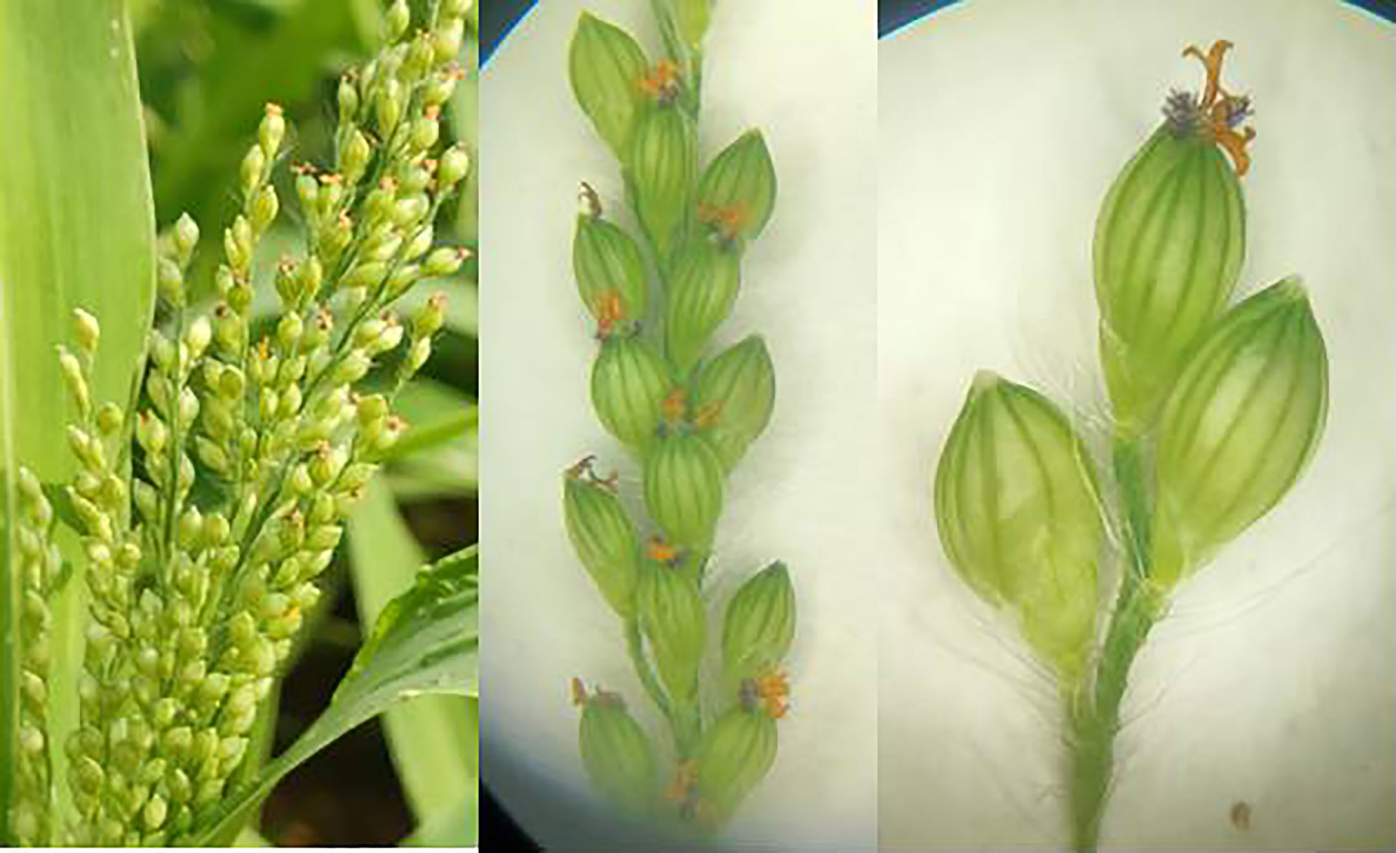
Blooming in browntop millet.

Two types of inflorescence, *viz.*, open and compact, are present (**Figure 15**). It has a branching panicle. The flower is bisexual with three anthers. In the center of the leaf blade, an evident vein is noticed in some cultivars (Basappa et al., 1987). The flowering is basipetal. On the fourth or fifth day of flower initiation, maximum flowering occurs (**Figure 16**). The research on browntop millet is scanty, especially on floral biology and anthesis in this crop. The contact method of crossing is employed in browntop millet, but the success rate of true hybrids is meager. To the best of the authors’ knowledge, there are no reported attempts on crossing techniques in browntop millet.

## Conclusion

Nature has favored a high magnitude of self-pollination in small millets, making the process of generating variability arduous. The intricate floral biology of millets hampers the process of artificial hybridization. Although, to some extent, few artificial hybridization methods have yielded successful results, it is essential to identify a method that produces 100% crossed seeds to harness the genetic potential of these mighty small millets.

## Author contributions

NC conceptualized the idea and compiled the draft. TEN, SB and GPS edited the manuscript. All authors contributed to the article and approved the submitted version.
